# Puberty and Pubertal Growth in Healthy Turkish Girls: No evidence for secular trend

**DOI:** 10.4008/jcrpe.v1i1.16

**Published:** 2008-08-02

**Authors:** Rüveyde Bundak, Feyza Darendeliler, Hülya Günöz, Firdevs Baş, Nurçin Saka, Olcay Neyzi

**Affiliations:** 1 Istanbul University, Istanbul Faculty of Medicine, Department of Pediatrics, Pediatric Endocrinology Unit, İstanbul, Turkey; +90−212 525 25 15+90−212 631 39 97bruveyde@istanbul.edu.trİ.Ü. İstanbul Tıp Fakültesi Çocuk Sağlığı ve Hastalıkları Anabilim Dalı, 34390 Çapa−İstanbul−Turkey

## Abstract

**Background**: Assessment of pubertal stages should be related to updated and reliable referance data from the same background population.

**Objective**: The aim of this study was to provide normative data for the onset and tempo of puberty in Turkish girls and analyze the growth parameters in puberty.

**Methods**: The analyses are based on data that were collected and evaluated biannually on 1020 Turkish school children aged 8−18 years and a subsample of 101 girls who had reached final height (FH). The data were analyzed cross−sectionally in the total group and longitudinally in the subsample.

**Results**: Mean age and height (Ht) at onset of puberty were 10.1 ± 1.0 years and 141.7 ± 7.6 cm, respectively. Peak height velocity (HtV) was 8.5 ± 1.0 cm/year. Total pubertal height gain was 16.0 ± 3.9 cm. The duration of puberty was 4.9 ± 1.2 years. Age at menarche was 12.2 ± 0.9 years. Height at onset of puberty was positively correlated with FH (p < 0.0001). Body size (weight and height) at onset of puberty and weight and height velocity before the year of onset of puberty correlated negatively with age at onset of puberty (p < 0.05).

**Conclusion**: In conclusion, these results provide normative data for pubertal stages and growth parameters in girls in puberty. Height at onset of puberty is the most important determinant of FH. There is no secular trend for the onset of puberty. Weight does seem to affect the onset of puberty but not FH.

**Conflict of interest:**None declared.

## INTRODUCTION

Although the sequence of pubertal changes in adolescence is predictable, the timing of puberty is variable.(1, 2) Variations in pubertal timing depend on genetic and environmental factors.(3) In addition, secular trend appears to influence the physiological range in the timing of pubertal onset.(4) The results of a relatively recent epidemiological study (PROS study) from the USA show an earlier sexual maturation in girls, confirming previous findings.(5) This study caused much debate in the scientific literature, because the estimates of the ages of onset of puberty were considerably earlier than those reported previously, leading to a questioning of the validity of the ages customarily used to define precocious puberty.(6,7,8)

In clinical practice, assessment of pubertal stages in the individual child is useful, but should at all times be related to updated and reliable reference data from the same background population. Since secular trend in growth and in timing of puberty is a phenomenon observed world wide, national reference data need to be collected at regular intervals. (9, 10)

We therefore aimed to provide normative data for the onset and tempo of puberty in Turkish girls and to analyze the growth parameters in puberty. We also compared the findings with those of a previous study on children of a similar background(11) and tried to evaluate the effect of a secular trend, if any.

## MATERIALS AND METHODS

Data on height growth and pubertal development were collected between the years 1989 and 2002 by biannual visits to six primary and secondary level schools in Istanbul city. The children born in 1975 or in subsequent years were classified by socioeconomic level into groups in accordance with previously reported criteria.(12) Of a total of 1020 girls, 439 girls aged from 8 to 18 years evaluated as of high socioeconomic class (Class 1) were included in the present analysis. Class 1 denotes children of high socioeconomic class in which the mothers’ years of schooling are longer than 8 years, the fathers’ longer than 11 years and the father is either a businessman, a professional or a high official. Over time, measurements were repeated on these same children, but other children were also included in the study to provide adequate numbers for the older age groups. Thus, our sample consists of a mixture of children followed longitudinally over different periods of time.

Approval from the Ministry of Education was obtained for the study. Informed consent was obtained from the parents of each child included in the study.

Chronological age was computed from the birth date reported by the child and from the school files. In case of disagreement between these two sources, the child was not included in the analysis. Chronic or debilitating disease were also reasons for exclusion.

Chronological ages of the children were grouped according to age in years±3 months. Height (Ht) was measured in a standing position with bare feet, using a portable measuring device (Leicester Height Measure, Invicta Plastics, Ltd, UK). A portable scale sensitive to 0.1 kg was used for weight (Wt) measurements, which were conducted with children in minimal underclothes. All measurements were performed by either of the two trained technicians throughout the study. Ht measurements were repeated twice, and the mean value was calculated. In case of a discrepancy exceeding 0.3 cm, a third measurement was done and the mean of the two closest values was used. Interobserver error for height measurements was assessed by having the same two technicians, independent of one another, measure a random subsample of 30 children (15 girls and 15 boys). Intraobserver error was assessed by having each technician conduct two measurements performed 1 week apart on every child in another random subsample of 15 children (7 girls, 8 boys). Inter− and intra−observer measurement errors, expressed as the standard deviation of the differences between these duplicate measurements, were 0.25 cm and 0.21 cm, respectively.

Final height (FH) was defined as attainment of a height velocity (HtV) less than 0.5 cm/yr. Breast (B), pubic hair (PH) and axillary hair (AH) development stages were rated in accordance with the Tanner criteria by one observer (RB) throughout the study.^1^ Evaluation of pubertal stages was also done at 6 monthly intervals. Attainment of a breast stage of 2 was accepted as the onset of puberty. Duration of puberty was taken as the time period from attainment of a breast stage of 2 to final height.

HtV was calculated from the multiple measurements taken on each individual child. To analyze the data, the children were grouped by their breast stages; and height velocities for each breast stage group were calculated. HtV at a certain breast stage was designated as the annual velocity following the attainment of that breast stage.

In a subsample of 101 girls who have been followed longitudinally until they reached their final height, age of peak height velocity (PHV) for each subject was estimated by plotting the measurements of the child on the growth chart and by taking the midpoint age between the two successive measurements (those preceding and following the most rapid height growth) as the age of PHV. Body mass index (BMI) was calculated as weight (kg)/height (m2).

The data were entered in a FoxBase program and analyzed by using SPSS−PC. Linear correlations were used to define the relationships between various parameters.

## RESULTS

**Total group:** Mean age of onset of puberty was 10.1±1.0 yrs for the total group of 439 girls. Age of onset of pubic hair was 11.0±1.0 yrs and that of axillary hair was 11.6±1.0 yrs. Mean age of menarche was 12.2±0.9 yrs (range 9.6−14.0 yrs). Onset of pubic hair development was earlier than breast development in 21.5% of the girls, breast development being the first sign of puberty in the remaining 78.5%. Age of percentiles at attainment of stages of pubertal development are given in Table 1. Relationships  between breast stage and weight, height, height velocity, weight velocity (WtV) and BMI are shown in Table 2. As seen in the Table, maximal HtV and WtV were observed after breast development reached stage 2.

The subsample of longitudinally followed girls: In the subsample of 101 girls who were followed longitudinally to final height, mean age at onset of puberty was 10.6±0.9 yrs (range: 7.5−12.3 years). Mean Ht at onset of puberty was 144.9±6.8 cm at a mean velocity of 7.4±1.5 cm/yrs. These values were similar to those of the total group. HtV before the year of onset of puberty was 6.2±1.2 cm/yrs and WtV before the year of onset of puberty 4.6±2.8 kg/yrs. The auxological and pubertal characteristics of the subsample of 101 girls who were followed to final height are given in Table 3. Duration of puberty was 4.9±1.2 yrs (range 2.1−6.5 yrs), when estimated as the time period from B stage 2 to final height. The time period from B stage 2 to menarche was 1.8±0.6 yrs. Mean age of menarche was 12.2±0.9 yrs. Total pubertal height gain (PHG) was 16.0±3.9 cm (range 8−25 cm). Growth in height continued after menarche and mean gain in height after menarche was 6.4±2.7 cm (range 2.2−14.0 cm). PHV was observed at a mean age of 11.3±1.5 yrs (range 8−13.1 yrs), and its amplitude was 8.5±1.0 cm/yrs (range 6.7−10.5 cm/yrs). As expected, these values showed a wide range. Mean final height was 163.7±6.0 cm.

Age at onset of puberty correlated negatively with weight, height, WtV before the year of onset of puberty (p=0.001; r=−0.3) and HtV before the year of onset of puberty (p=0.01; r=−0.6). There were no significant correlations between age at onset of puberty and BMI, final height, total PHG, duration of puberty, and PHV. Age of menarche was negatively correlated with weight at menarche (p=0.001; r=−0.3), BMI at menarche (p=0.001; r=−0.3) and positively correlated with age at onset of puberty (p=0.002; r=0.5). Final height was correlated only with  height at onset of puberty (p=0.0001; r=0.8).

**Table 1:**
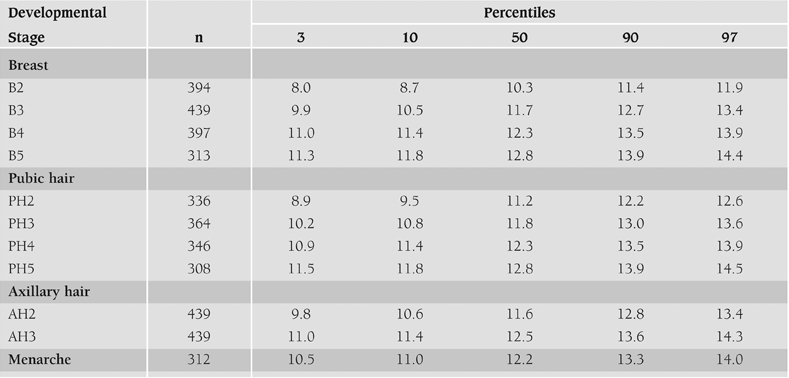
Median ages (years) and 3^rd^, 10^th^, 90^th^, 97^th^ percentiles (ages in years) at attainment of stages of pubertal development in the total group

**Table 2:**
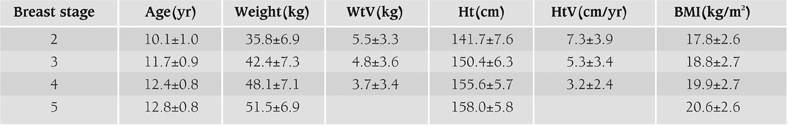
Results by breast stages in the total group of girls (mean±SD)

**Table 3:**
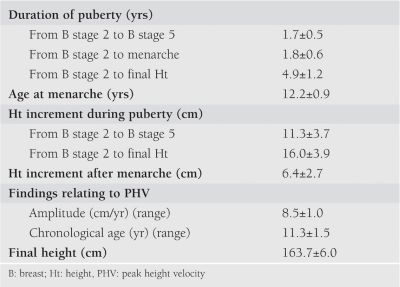
Auxological and pubertal characteristics of the girls followed to final height (n=101) (mean±SD)

## DISCUSSION

This study provides references for pubertal stages in Turkish girls which can be used for clinical purposes. Stages of secondary sexual maturation can be recorded from direct examinations, inspection of photographs, and self−assessments. Mailed questionnaires have also been used. It is usually assumed that data from direct examinations have greater precision and validity than those obtained by other procedures. The information about the tempo of puberty can only be obtained by longitudinal studies. The longitudinal assessment of puberty in girls performed by Marshall and Tanner is one such example.(1) On the other hand, the difficulties in producing normative data in a developing country are well known and only a sample from the upper strata of the population will suit the recommendations of the World Health Organization (WHO) for obtaining reference values.(13, 14, 15) We believe that the reliability of the data in our study is high, due to the longitudinal design of the study and also due to the fact that the subjects consisted of the upper strata of the population. It must be added that the mixed population of Istanbul city is quite representative of the whole of Turkey. (16, 17)

Relatively few data are available on the accordance between B and PH stages during puberty. Traditionally, breast development is accepted as the initial event in pubertal development in girls. However, PH was observed to occur before breast development in 21.5% of the girls in our study. This finding was 10% in Dutch girls and approximately 30% in a British study.(1, 18)

The definition of precocious puberty is based on the age of occurrence of B or PH development in healthy girls in the population. Third percentile (P3) or −2 SDS are used as cut−off ages. The P3 age for B2 is 8.0 years (Table 1). In Dutch girls P3 age for B2 is 8.2 years, a cut−off age close to our findings.(18) Eight years is generally and internationally used as the cut−off age for the definition of precocious puberty in girls. Our findings suggest that this cut−off age can be continued to be used for Turkish girls.

In a previous study on the sexual maturation in Turksh girls born in 1955−1960 mean age for onset of breast development was 9.8 years, and 10.3 years for PH development. Mean age of menarche was 12.4 years.(11) These findings, as compared to this present study, suggest that there appears to be no secular trend for earlier maturation in girls in our population in the past few decades. One explanation for the absence of a secular trend in pubertal development may be that the children in both studies were taken from amongst the high socioeconomic level population. The influence of socioeconomic status, a likely marker for better health and nutrition, was illustrated by a recent study from India that reporteda mean age of menarche of 12.1 years for well−off girls and 15.4 years for underprivileged girls.(19) A few countries have examined secular trends for puberty and menarche over the latter half of the 1900s. Our study is in agreement with most studies from Northern European countries reporting no secular trend for girls.(18, 20)

This study also presents stages of pubertal development percentiles. The 50th percentile values in our study indicate younger ages as compared to Stockholm girls.(10) There is one study in Turkish girls that has investigated age of menarche in an urban area in Turkey and has found that age of menarche was 12.7±1.1 years in high socioeconomic level girls.(21) Although this age is higher than the mean age in our study, it should be emphasized that that study was done by questionnaire method.

To our knowledge, this is the first longitudinal study to report the relationship between the pubertal stages and growth parameters in Turkish healthy girls (Table 2 and 3). HtV and WtV were highest after attainment of breast stage 2. The different growth parameters such as age at PHV, magnitude of PHV and FH in longitudinally followed girls in our study were within the ranges of corresponding values reported in European and American studies.(22, 23, 24) The attainment of final height depends on several factors at interplay. One factor may be the differences in height at onset of puberty. The positive correlations found in our study between height at onset of puberty and final height indicate that the smaller the child is at the onset of puberty, the smaller the final height will be, a finding which has been reported in other studies as well.(25) This finding points to the importance of height at onset of puberty for the prediction of FH. Another factor affecting FH may be the total PHG. However we found no correlation between total PHG and FH, in compliance to other studies.(26)

It has been shown in a number of studies that age at onset of puberty is not correlated to the duration of puberty,(1, 2) a finding which has been confirmed in our study as well. This implies that the duration of puberty does not change with respect to the age at onset of puberty.

It has been reported in many studies that there is no difference in FH between early and late maturers.(27, 28, 29) In our study, in compliance with the study on Swiss children, (23) no correlation was evident between age at onset of puberty and PHV, total pubertal height gain or FH. Thus it may be concluded that height attained at onset of puberty is the main factor affecting FH.

It is also noteworthy that weight, height at onset of puberty and HtV and WtV before the year of onset of puberty are negatively correlated to age at onset of puberty, implying that the heavier the child is earlier is the puberty. This relationship between body size and the timing of puberty has been shown in several studies, mostly in girls.(30, 31)

In conclusion, there seems to be no secular trend for the onset of puberty in our population for girls. The same was shown to be true for Turkish boys.(32) Body size at onset of puberty does seem to affect the age of onset of puberty, namely, the heavier the girl is at onset of puberty, the earlier the puberty will be, but this does not appear to affect the final height. Similarly, heavier girls had menarche at earlier ages. This is the first longitudinal study to report the relationship between the pubertal stages and growth parameters in Turkish healthy girls. As also confirmed by the findings in this study, the most important determinant of final height appears to be height at onset of puberty.

**Table 1:**
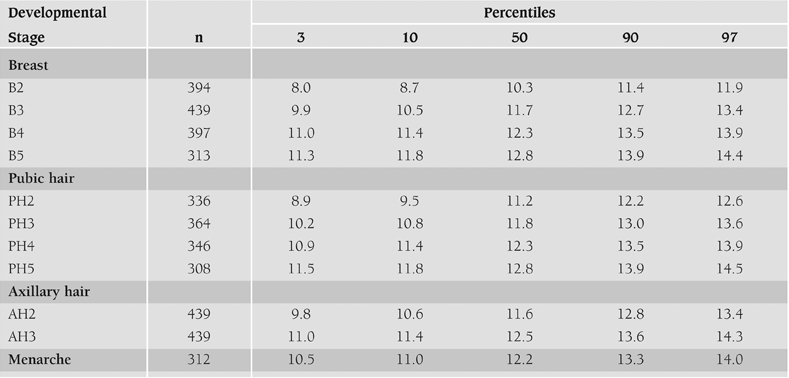
Median ages (years) and 3^rd^, 10^th^, 90^th^, 97^th^ percentiles (ages in years) at attainment of stages of pubertal development in the total group

**Table 2:**
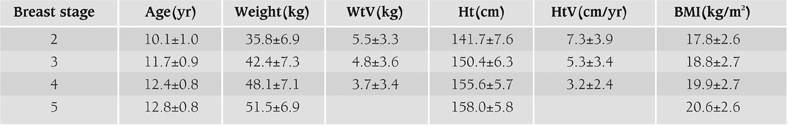
Results by breast stages in the total group of girls (mean±SD)

**3:**
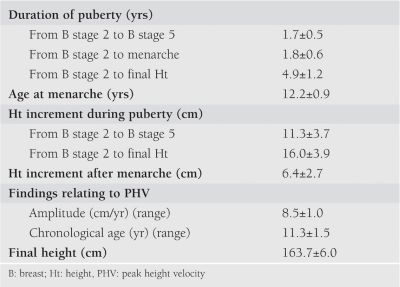
Auxological and pubertal characteristics of the girls followed to final height (n=101) (mean±SD)
